# The experiences of detained mental health service users: issues of dignity in care

**DOI:** 10.1186/1472-6939-15-50

**Published:** 2014-06-27

**Authors:** Mary Chambers, Ann Gallagher, Rohan Borschmann, Steve Gillard, Kati Turner, Xenya Kantaris

**Affiliations:** 1 Faculty of Health, Social Care and Education, Kingston University and St. George's, University of London, London, UK; 2 Faculty of Health and Medical Sciences, University of Surrey, Guildford, UK; 3 Institute of Psychiatry, King’s College, London, UK; 4 Institute of Population Health Research, St. George's, University of London, London, UK

**Keywords:** Detained, Mental health, Service users, Dignity, Ethics, Nursing, Respect

## Abstract

**Background:**

When mental health service users are detained under a Section of the Mental Health Act (MHA), they must remain in hospital for a specific time period. This is often against their will, as they are considered a danger to themselves and/or others. By virtue of being detained, service users are assumed to have lost control of an element of their behaviour and as a result their dignity could be compromised. Caring for detained service users has particular challenges for healthcare professionals. Respecting the dignity of others is a key element of the code of conduct for health professionals. Often from the service user perspective this is ignored.

**Methods:**

This paper reports on the experiences of 19 adult service users who were, at the time of interview, detained under a Section of the MHA. These service users had experienced coercive interventions and they gave their account of how they considered their dignity to be protected (or not), and their sense of self respected (or not).

**Results:**

The service users considered their dignity and respect compromised by 1) not being ‘heard’ by staff members, 2) a lack of involvement in decision-making regarding their care, 3) a lack of information about their treatment plans particularly medication, 4) lack of access to more talking therapies and therapeutic engagement, and 5) the physical setting/environment and lack of daily activities to alleviate their boredom.

**Conclusions:**

Dignity and respect are important values in recovery and practitioners need time to engage with service user narratives and to reflect on the ethics of their practice.

## Background

Admitting an individual to a clinical environment involuntarily can be distressing for all involved. For the service user, it will usually be perceived as unnecessary and may result in physical resistance [1]. Being detained under the MHA impinges upon individuals’ freedom and has the potential to diminish service user dignity. Dignity is a value that assumes a central role in the Universal Declaration of Human Rights [2], of which Article 1 states that, *"All human beings are born free and equal in dignity and rights"*. Dignity is also a central value in codes of professional conduct. The International Council of Nurses’ Code [3], states that, *"Inherent in nursing is respect for human rights including cultural rights, the right to life and choice, to dignity and to be treated with respect".* Dignity is also a contested concept. Macklin (2003) [4] argues that, unlike the principle of autonomy, dignity is *‘a useless concept in medical ethics’* as it is vague and imprecise. Despite critiques from Macklin and other philosophers, there has been considerable activity regarding the development of dignity declarations, policy, scholarship and research. Much of this activity appears to have been prompted by concerns about care deficits [5]. According to Shotton and Seedhouse (1998) [6], we lack dignity when we find ourselves in inappropriate circumstances, when we are institutionalised, where we feel foolish, incompetent, inadequate or unusually vulnerable.

The Mental Health Act (MHA) was first introduced in the UK in 1959, (with major revisions in 1983 and 2007). The purpose of the current Act is to ensure the safety of those with mental health problems and that of others in the community. The MHA makes provision for compulsory or involuntary psychiatric care if a person is deemed to be experiencing a mental health condition that is of a nature and/or degree deemed appropriate for care and judged necessary to safeguard the health and safety of the individual or the protection of others. The majority of individuals currently accessing mental health services are admitted to hospital, under a Section of the MHA [1]. The implementation of the Act highlights three key ethical issues: 1) the person’s right to receive medical treatment, 2) their right to liberty and dignity, and 3) the protection of the public. The constraints on the provision of care in inpatient settings including the staffing and resources must be acknowledged and indeed that service users may have been very unwell upon admission and not necessarily able to ‘choose interventions’. The service users’ right to receive medical treatment and their safety ensured has already been outline however in the ‘call of duty’ , under some circumstances such as where coercive interventions are used, these might compete with the preservation of dignity from the perspective of the service user.

Treatment can be provided to mental health service users without their consent if it is judged by professionals to be in their best interest and supported by the principle of beneficence. The ability to contribute to the decision-making process about their plan of care, the opportunity to express autonomy, to give and/or withhold consent freely and the right to confidentiality is sometimes denied to those detained under the MHA [7]. The result of such denial can be a sense of powerlessness and/or helplessness which can impinge upon service user self-confidence and self-esteem, thereby hindering recovery [8,9]. Chochinov’s research relating to dignity therapy demonstrates that where patients lack dignity they may, quite literally, lose the ‘will to live’ [10]. Strengthening service users’ dignity can increase confidence and satisfaction with care, whereas disrespecting dignity can result in deterioration in both physical and mental health [11]. When service users are admitted under a Section of the MHA their levels of distress can manifest itself into an act of physical behaviour [1] that can result in danger to themselves and/or others in their immediate environment. When such behaviour is experienced by nursing staff coercive forms of interventions such as control and restraint, rapid tranquilisation and/or seclusion may be used. Being exposed to such interventions is an affront to service user dignity [12]. The Care Quality Commission (CQC) (2010) [13] has a statutory duty to monitor how service providers exercise their power under the Act and to provide a safeguard for service users. A further safeguard are the National Institute for Health and Clinical Excellence (NICE, 2005) [14] guidelines on the techniques to be used by professionals in the management of violence and aggression which stipulates that coercive interventions should be used only as a last resort.

This study aimed to explore the service user experience of detained care. In particular, service users were asked to reflect upon how their dignity and respect was considered when sectioned and subjected to coercive interventions and asked to reflect upon answers on issues of major significance and/or importance to them.

## Methods

### Setting and participants

The project took place in 3 mental health hospitals of similar size and nature in the South East of England and involved 19 service users who were detained under Section 3 of the UK MHA (1983; 2007) at the time of data collection. Service users were detained between 2 weeks to 2 months at the time of being interviewed.

A purposive sampling technique was used; purposive sampling is sampling with a purpose in mind where the researchers have one or more specific predefined groups or types of people that they are seeking. Purposive sampling can be very useful for situations where you need to reach a targeted sample quickly and where sampling for proportionality is not the primary concern. With a purposive sample, you are likely to get the opinions of your target population [15]. The service user participants were identified by both the ward manager of their inpatient ward and consulting Psychiatrist as primarily having capacity and secondly as someone who would be interested in discussing their experiences of being detained, (from admission to discharge). Potential participants were given the study information sheet by the person who would interview them; this would either have been a service user researcher or a clinical psychologist all of which were members of the research team. The participants were given the opportunity to ask questions and to confirm their interest in participating in the study. Participants were also informed that their engagement in the project was entirely voluntary and that if they wished to withdraw at any time this would not impact upon their access to and receipt of services and clinical support.

Those participating had all experienced some form of coercive intervention such as control and restraint, rapid tranquillisation and/or seclusion (according to the ward manager on their inpatient ward). Of the 19 participants, 12 (63%) were male and 7 (37%) were female; 7 (36%) were black British, 10 (52%) were white British and 2 (12%) were of other ethnic origin. Their mean age was 35 (SD 10.0, range 19–53). All service users were resident in an acute admission ward, a psychiatric intensive care ward (PICU) or a forensic inpatient ward and had sufficient symptom stability according to the responsible Psychiatrist and ward manager, to participate in an hour long interview on one occasion.

### Data collection

Interviews were conducted over a six-month period. The semi-structured interviews explored in detail, service users’ experiences of detention and of coercive interventions. The interviews were undertaken by a combination of three service user researchers i.e. researchers with personal experience of inpatient mental health treatment and of being detained under the MHA, and a clinical psychologist all of which were part of the research team. All the interviews were conducted in private in a quiet room in their inpatient ward. As the focus of the study was on capturing and understanding the lived experience of detained care, the input of service users as members of the research team was considered to be important and beneficial due to their greater sensitivity and empathy of such issues [16]. The interviews took place in the clinical environment in which the service users were detained. An interview schedule served as a guide for each interview and to ensure consistency of questioning. Examples of questions include, why were you detained/what were the circumstances surrounding your detainment; describe your experience of being admitted to hospital; were you involved in your care plan, if so, how did you contribute? Inexperienced interviews were given training to conduct the interviews as appropriate.

The interviews were audio-recorded and subsequently transcribed verbatim. The duration for the interviews varied from 45 to 60 minutes. Each participant was assigned a code number for the interview to ensure anonymity. Participants were informed that the staff team and/or police would be informed of anything that constituted a danger and/or referred to anything that might be criminal in nature.

### Data analysis

The data from the interview transcripts were analysed using an (inductive), thematic approach whose purpose was to describe and draw inferences and/or conclusions, patterns and/or trends about the characteristics of communications major headings which refer to the producers or causes of content, and the audiences and consequences of content [17]. This relational analysis explored the spectrum of possible relationships between analytical themes within the data and the relationships among the concepts in the data. Relational analysis is a technique used that explores relationships of identified concepts in a text. This type of research takes non-statistical methods to explore the relationships of concepts found in groups of text. In other words, the focus of relational analysis is to look for semantic, or meaningful, relationships within the content of the data [18]. Any passages and/or situations from the transcripts of interest and importance together with commonalities were captured across the interviews and given a short label or description. The data were then categorised according to 5 broad key themes and their sub-themes. No new key themes or sub-themes emerged after analysing the first 10 interviews. It should be noted that the themes were not predetermined as the study was exploratory.

### Ethical considerations

A favourable ethical opinion was obtained from local Research Ethics Committee (06/Q0803/198) and the Research and Development (R&D) committee of each participating hospital. To comply with the conditions of ethical approval, enhanced support was made available to the service user researchers if during interviews if required. Post interview briefings took place as part of the on-going supervision process.

## Results

The overarching principal theme that emerged was a desire to be treated with dignity and respect by staff. Five other key themes were identified. Table 1 summarises the key themes and their associated sub-themes. The themes are presented with supporting quotations from the service user participants. The quotations included illustrate and validate interpretations [19].

**Table 1:** Key themes and their sub-themes

***Principle Theme: Dignity and Respect***	
***Key themes***	***Sub-themes***
***‘Heard’ by staff members***	*Powerlessness and lack of autonomy*
	*Self-worth*
	*Staff/service user relationships and staff attitudes*
	*Stigmatisation*
***Involvement in decision-making regarding their care***	*Future prospects*
	*Powerlessness and lack of autonomy*
	*Self-worth*
***Information about their treatment plans (particularly medication)***	*Powerlessness and lack of autonomy*
	*Self-worth*
	*Staff/service user communication and staff attitudes*
***Access to more talking therapies and therapeutic engagement***	*Restraint and feelings of helplessness*
	*Seclusion/timeout*
***Physical setting/environment and Daily activities (to alleviate boredom)***	*Physical surroundings*
	*Feelings of imprisonment*

The findings presented and discussed below are the perceptions, reports, views and interpretations of the participants and were not independently observed by the interviewees. The connection between the overarching theme of dignity, the other broad themes and their subthemes were relationships/associations that the authors identified during the content analysis of the reports from the service users. The study aimed to explore the service user experience of detained care. In particular, service users were asked to reflect upon how their dignity and respect was considered when sectioned and subjected to coercive interventions. The focus of the study was on capturing and understanding the lived experience of detained care; at no time were the service users asked directly if their dignity was impacted upon.

### ‘Heard’ by staff members

In some way or another all interviewee spoke of a sense of powerlessness and lack of autonomy whilst detained, which was deemed to diminish their sense of self-worth. This alongside reported poor relationships with staff and staff attitudes led to the feeling that they were being marginalised for having a mental illness by staff and society. These feelings were accompanied with frustration at not being treated as human beings. There does seem to be a conflict between taking care of service user autonomy and the principle of beneficence. Positive elements of the staff and service user relationships, communication and staff attitudes were also captured during the interview process. The following quotations illustrate this theme and its sub-themes.

I found that they (staff) had confidence in me and I had confidence in myself, so I could go out on the grounds. I knew that they (staff) could trust me and I could trust them. So there was this bond of trust which is quite imperative… when you speak to maybe your named nurse, he or she would literally tell you things that can attribute to positive things in your mental health (Participant 6)

… we can become sort of entities of psychological habits, and they can put us not as a human being but as a series of quirky behaviours which they can sort of diagnose in their own ways, you know? … forever sort of speaking abruptly and saying "You’re not answering me correctly, go to your room and have time out for fifteen minutes", and you felt like a naughty child, you know? It’s sort of in your mind that you’ve got to watch your p’s and q’s (Participant 13)

Service users reported that they felt marginalised and discriminated against not only in the ‘outside world’ but within the ward by staff. Adults with mental health problems are one of the most socially excluded groups in society [20]. Participants reported that they sometimes felt marginalised and/or discriminated against by members of staff and also humiliated and embarrassed. The following quotes demonstrate these feelings.

So it’s a double-edged sword when you’ve got mental health problems. You’re labelled. At home you’ve got a label, and you’re labelled in the system, so there’s not a great deal of dignity afforded to you. Yeah, and also everyone like your relatives knows you’ve been in here, oh, it’s happened again, and you are the sort of black sheep of the family, so you’ve got lots of things on your head really. The label sticks, it really sticks (Participant 13)

They’re the nurses, we’re the mentally ill, and maybe that’s it. Basically, we’re supposed to be mad, and they’re supposed to be perfect, you know what I mean, and pristine. Play the game and get on with it (Participant 16)

There is a somewhat complex relationship between detained service users and staff members involving staff attitudes and what information is communicated to those detained about their section, care and/or treatment. It may be a challenge to find a reasonable balance between closeness and distance in interactions with service users however, mistrust and a lack of confidence seems to have arisen in communication between service users and staff according to interviewees in this study. The relationship(s) and/or communication issues may hamper or promote service users’ dignity with regard to their worth and value as individuals. This is illustrated by the following quotes.

But overall, they’re together, kind of, and they do things against you. One thing that needs to change is the way that they feel that they’re higher than you, and that they have a duty of care over you, because most of them don’t even care, you know? They don’t seem to have much love or respect for you (Participant 12)

### Involvement in decision-making regarding care

Although participants were detained under a Section of the MHA, it is important that as soon as their mental state permits they are able to input into the decision-making process regarding their care. When dignity is present people feel in control, valued, confident, comfortable and able to make decisions for themselves. With respect to service user input into decisions regarding care, the data indicate a mixed and inconsistent picture with views ranging from adequate information to no involvement in decision-making. Some participants described being coerced into taking medication and having ‘no say’ in treatment. Participants spoke of a sense of powerlessness and lack of autonomy whilst detained suggesting a conflict between service user autonomy and the principle of beneficence. Data showed that service users felt ignored and not listened to and that needs, wants and feelings were disregarded and overlooked. Also highlighted were the differences in the approach between doctors and nurses, with the former being more ‘directive’ and the latter described as ‘psychologically intimidating’.

The data suggests a mixed picture regarding service user views of the different professional groups. Service users appeared sensitive to staff behaviours and attitudes that may not be in harmony.

*Definitely restricted* (with regards to medication treatment), *but coerced into taking medication. Cos nothing changes, when you ask for something to change, like medication, nothing changes until they’re ready to change you. (Participant 1)*

Um, yeah, I was given enough information. I was told, you know, what might happen and there are medications for the side effects (Participant 9)

They’ll take no notice of what you say (in ward rounds). They’ll pay no attention to what you have said. The nursing staff treats everyone the same but the doctors do not. I’ve seen him look at her, obviously he feels nothing but contempt for her. The doctors like for you to think that they’re kind, considerate, thoughtful, understanding, loving. Providing that you go along with it and say, oh doctor, you’re wonderful, you’re so kind to me, what would I do without you? (Participant 17)

Several interviewees reported that they felt that their future was tainted due to sectioning and detainment therefore views/outlook about their chance and/or vision of their future was pessimistic.

Cos they’re always saying they want to get us back to work and things …But it’s hard in a place like this; you just become institutionalised and there’s no goals to set (Participant 7)

### Information about treatment plans (particularly medication)

Data show that service users wanted information about medication and its potential side effects.

No-one was explaining to me what was happening. Uh, it was just given to me (medication); nobody did sit and discuss it with me (Participant 14)

### Coercive interventions and alternatives

Participants described having experienced coercive practices and/or having witnessed them happen to others. Coercive practices, whilst seen as necessary in some situations, appeared to have physical and psychological consequences for the service user and were viewed as adverse. Service users reported that staff seemed to know what they needed to do to avert untoward incidents i.e. hostile verbal and/or physical behaviour but did not act accordingly. Explanations suggested by participants were lack of time, did not persevere to meet service users’ needs, could not stand any commotion, and consequently did not act in the service users’ best interests.

In care situations, dignity can be promoted or diminished. Alternatives to coercive practices may help service users feel, think and behave in relation to their worth and/or value of themselves and others. Participants highlighted the need to find alternatives to coercive interventions e.g. counselling and timeout. In this context timeout was referring to being given personal space in a ‘quiet room’.

I don’t think I got proper counselling to begin with, in the very instance that I was admitted. And this is probably what is so crucial, imperative, to get one-to-one counselling and really talk it through… I think counselling’s the most essential thing, you know? Without it, patients are lost. ‘Cos then they’re gonna be kind of lost, they’re gonna be constantly lost, and then pumped with more medication that they don’t need. (Participant 6)

"Medication! Medication!", right? I think on this ward, not straight away medication, sort of like "calm down" first, instead of always giving people medication. Not talk things through, but they sort of like calm things down, or try to anyway (Participant 10)

Service users either described being put in seclusion or having witnessed this happen to others.

When they dragged me forward, one of them had to drag me by my trousers… And they pulled them down, and left them. That’s out of order. It depends how it happens, some of them can be really embarrassing. Like me, I was getting dragged along in my underpants (Participant 12)

As with seclusion service users either described being restrained physically and/or chemically or having witnessed this happen to others.

Well, the scariest time was the first time, I was just petrified. And then the team came in and they held me down and um, they gave me an injection, and I was very scared (Participant 16)

It depends how they do it. Only giving the patient medication does not include being especially cruel to them. All they’ve got to do is hold them down and give them medication. Some of them can be cruel, you know? They pull their clothes around their neck and they’re almost strangling them, and they hold their hand behind their back and it’s cruel and they’re squeezing their arms. Some of them can be very cruel…. Some of them take unfair advantage. (Participant 17)

### Feelings of imprisonment and physical environment

Some service users compared being detained to that of imprisonment. They reported feeling powerless with no autonomy and that their actions were restrained and controlled. Lack of privacy, being under constant supervision and all wants and/or needs e.g. ground leave, had to be negotiated with staff as demonstrated in the examples that follow.

There’s less, uh, restrictions in prison, you know? Like, we feel like we’re in prison but they call it a hospital (Participant 3)

It would be nice to have some freedom and be treated normal. I mean, they’ve got windows here and obviously you can see outside and you’ve got air coming in and everything, but I think people just need to be outside every now and then (Participant 12)

Participants also commented on the physical characteristics of their environment/clinical setting. Environmental issues can threaten dignity in wards include overcrowding, poor staffing (levels and quality), and impoverished or unclean environments [21]. This includes the spatial and material aspects of the service users’ ward.

Space I think is really important; everyone requires their own space, even down to their dormitories (Participant 6)

Yeah, it’s not a bad ward at all and the effort that they’ve been putting into the ward with regards to decorating and stuff… they’re making it a more cheerful and homely place (Participant 9)

## Discussion

The objective of this paper was to explore the experiences of service users detained under the United Kingdom Mental Health Act 1983/2007. The data suggests mixed views and experiences encompassed in the key themes and sub-themes that emerged ranging from positive attitudes to those of undignified treatment. There was some strain concerning the positive relationships with staff, general care and/or treatment and/or involvement in decision-making regarding care planning. This finding is significant in terms of the way that mental health professionals work with service users and the important effect on their recovery [22]. Building and establishing effective and trusting relationships with service users promotes and supports recovery and enables involvement and control over their ‘conditions’ , treatment and care. It is important that staff members hold positive attitudes when caring for distressed service users to help them understand their situation and get the best possible outcome [23]. Negative staff attitudes may lead to further frustration on the part of service users resulting in acts of physical aggression and culminating in coercive interventions [24]. Conversely, some service users reported instances and encounters of disrespect and undignified treatment and/or care that impinged upon their dignity. These findings support previous research [8,9], by suggesting that feelings of powerlessness, helplessness and stigma, impinge on the service users’ sense of dignity.

Shared decision-making is a fundamental component of recovery and active service users participation in care and treatment planning is a critical part of rehabilitation. Providing the opportunity for service users to choose interventions that fit personal preferences increases the likelihood that these interventions will have personal meaning, enhance satisfaction, improve quality of life (QoL) and therefore aid recovery. Our data suggests that when service users are detained, participants’ perceive their right to opinions and judgments as diminished and their wishes and/or opinions not taken seriously. Service users reported feeling ignored, not listened to and their needs, wants and/or feelings being disregarded, overlooked and/or not acknowledged. Detained service users often feel stigmatised by their experiences of services and/or service providers; the stigma associated with mental illness is pervasive among healthcare workers and forms a real barrier to optimal recovery from the illness so health professionals have a responsibility to improve their own attitudes and behaviour towards people with mental illness [11].

Staff members did appear to apply the principle of beneficence, which is, prioritising doing good and the interests and well-being of service users. Nonetheless, service users reported negative feelings of being put into seclusion and being restrained consequently finding it difficult to empower themselves. To cease these practices, staff and service users alike would need to engage in relationships that include positive affirmations like respect. If staff feel that they understand service users better than the service users understand themselves it is difficult for service users to become autonomous, confident and/or empower themselves [25].

In a mental health practice environment, a denial of rights [26] has the potential to de-value and/or disregard the biography and capabilities of the service users. To overcome this, mutual encouragement and respect for the service user is required, as well as developing a sense of solidarity amongst staff members and service users in general i.e. a ‘working’ partnership. The ‘normalisation’ of doing ‘regular’ , simple daily activities with and/for service users may act as respite from an authoritative staff- service users relationship and may act to restore a measure of dignity.

Mental health practitioners seemed to focus on solving service users problems primarily from a biomedical point of view and the service users’ perspective, values, wishes and/or needs did not seem to be sufficiently considered. With their ‘superiors’ , professionals should build upon learning from their work and focus on complex decision-making within mental health practice. Enhanced awareness of how clinical decision-making can impact upon ‘self’ , service users, professionals and service delivery. There is a need for holistic care within institutions that have a violating culture [27,28]. Our data suggested that in certain situations mental health workers may have demonstrated ethically questionable behaviours when interacting with detained service users. This may be due to the nature of the intense and demanding clinical environment [27,28].

Recovery-orientated ways of thinking i.e. social and/or clinical outcomes and participation are required. It is possible that the number and impact of violent incidents i.e. physical acts of aggression and/or hostility to self and/or others in acute mental healthcare settings could be reduced by the appropriate use of advance directives as part of the Care Programme Approach (CPA) [29] and consequently help reduce the use of coercive interventions.

The results of this study represent personal experiences which were transformed from the unspoken to the explicit. The findings could be transferred to other similar mental health inpatient settings within the NHS or social services and the wider health and social care environments across Europe and beyond as this is a universal issue [24]. Clinical settings could be considered typical and the sample representative of those detained service users detained under the MHA [8,9]. The data were gathered via interviews, some conducted by service user researchers that took place during the participants’ period of detention thus avoided the pitfalls of retrospective research, such as recall bias and accuracy [27]. However, we are mindful that the lack or limited autonomy of service users, service users being afraid that information will be revealed to staff members, service user involvement in the ‘situation’ (therefore lacking distance) may have impacted on the an objective picture of detention and the service user experience.

## Conclusions

This paper has reported on a fundamental value in mental health care - the dignity of detained service users. Our findings suggest that despite the rhetoric regarding service user involvement there still remains the need to address a range of issues within mental health nursing that could threaten dignity in care settings [28]. The data suggests that the attitudes and behaviour of staff members (people) are key to the enhancement or undermining of dignity in this challenging aspect of mental health practice. The physical environment and organisational culture (place) also needs to be considered if respect for service user dignity is to be operationalised and embraced throughout [29]. Both people and place need to convey the worth or value of all service users, particularly, when they are at their most vulnerable.

## Competing interests

The authors declare that they have no competing interests.

## Authors’ contributions

MC and SG designed the study and MC obtained the funding. RB and KT provided input throughout the study to assist in refining the process and interviewed the service users. All authors analysed the data. MC and XK drafted the manuscript. All authors read, made significant contributions and approved the final manuscript.

## Pre-publication history

The pre-publication history for this paper can be accessed here:

http://www.biomedcentral.com/1472-6939/15/50/prepub
